# The Brain-Derived Neurotrophic Factor Val66Met Polymorphism Is Associated With Female Obsessive-Compulsive Disorder: An Updated Meta-Analysis of 2765 Obsessive-Compulsive Disorder Cases and 5558 Controls

**DOI:** 10.3389/fpsyt.2021.685041

**Published:** 2022-01-12

**Authors:** Yanan Shang, Na Wang, En Zhang, Qian Liu, Hengfen Li, Xiaofeng Zhao

**Affiliations:** ^1^Department of Psychiatry, First Affiliated Hospital of Zhengzhou University, Zhengzhou, China; ^2^Department of Neurological Rehabilitation, Second Affiliated Hospital of Zhengzhou University, Zhengzhou, China; ^3^Department of Psychiatry, The Fourth Hospital of Wuhu City, Wuhu, China

**Keywords:** Val66Met polymorphism, brain-derived neurotrophic factor (BDNF) gene, obsessive-compulsive disorder (OCD), meta-analysis, female

## Abstract

**Objective:** Accumulated evidence has implicated that brain-derived neurotrophic factor (BDNF) gene polymorphisms play a role in the etiology of obsessive-compulsive disorder (OCD). A single nucleotide polymorphism in the coding exon of the BDNF gene at position 66, Val66Met (rs6265), is found to be associated with OCD in different populations, but results linking Val66Met with OCD have been inconsistent and inconclusive. In our study we performed a meta-analysis to further examine whether rs6265 genetic variants are involved in the etiology of OCD.

**Methods:** By searching databases, relevant case-control studies were retrieved; using established inclusion criteria, we selected eligible studies for analysis.

**Results:** Thirteen studies were identified that examined the association between the rs6265 polymorphism and OCD. After statistical analyses, no significant association was found between the rs6265 polymorphism and OCD (OR = 1.07, 95% CI = 1.00–1.15, *P* = 0.06 for genotype; OR = 1.06, 95% CI = 0.98–1.15, *P* = 0.15 for allele). However, in gender-specific analysis, female Val carriers might be a risk factor for OCD (OR = 1.36, 95% CI = 1.03–1.80, *P* = 0.03 for genotype; OR = 1.15, 95% CI = 1.01–1.32, *P* = 0.04 for allele).

**Conclusion:** Our updated meta-analysis suggests that female carriers of the Val66Met BDNF polymorphism might be more suspectable to develop OCD.

## Introduction

It has been demonstrated by twin studies that genetic factors play a key role in the etiology of obsessive-compulsive disorder (OCD) (1). While these genetic factors account for 27–65% of the variance in this disorder (1), its etiology has not been clearly elucidated in the past decades.

The brain-derived neurotrophic factor (BDNF) gene is considered to be one of the candidate genes of psychiatric disorders, as it is widely expressed in the brain and plays a major role in neurogenesis, neuronal growth, maturation, survival, synaptic plasticity, and microarchitectural integrity (2, 3). BDNF is also considered to play a role in regulating mood and behavior, and abnormalities in the expression of this gene have been involved in major anxiety disorder, depressive disorder, and OCD. Preclinical studies have shown that levels of BDNF are drastically decreased in the serum and brain tissue of rats with obsessive-compulsive disorder compared to a control group (4). In a clinical study, Maina was the first to demonstrate that levels of serum BDNF were significantly decreased in OCD patients compared to normal controls (5). Following that study, Wang et al. (6) suggest that BDNF is involved in the etiology of OCD, and may be a peripheral marker indicating neurotrophic impairment in OCD similarly, Suliman et al. demonstrated that peripheral BDNF levels in OCD patients were significantly lower compared with that of normal controls with this data, it can be speculated that the BDNF gene is involved in the etiology of OCD.

Researchers have extensively focused on the BDNF polymorphism, Val66Met, also known as G196A or rs6265 (7). This polymorphism influences the activity of BDNF and impairs BDNF signal transduction, and therefore may be a possible candidate gene involved in the etiology of OCD (7). While previous evidence suggests that the rs6265 polymorphism is involved in the etiology of OCD, the outcomes of such studies have been inconsistent and even contradictory. Findings from the latest meta-analytic investigation by Wang et al. in 2019 provide little support for the Val66Met variant as a predictor of OCD (8). In order to derive a more putative conclusion, an updated meta-analysis was performed to study the connection between the rs6265 polymorphism of the BDNF gene and the risk of OCD.

## Methods

### Inclusion Criteria

(1) All studies investigated the association between OCD and Val66Met, G196A, or rs6265 polymorphisms. (2) OCD was diagnosed according to the Diagnostic and Statistical Manual of Mental Disorders (DSM) or the International Classification of Diseases (ICD) and included DSM-III, DSM-III-R, DSM-IV, DSM-IV-R, and ICD-10. (3) The study had a patient group with OCD and a control group. (4) The study included samples from varied ethnic backgrounds. (5) The frequencies of each allele and genotype met the criteria for Hardy-Weinberg disequilibrium in both case and control groups.

### Search Strategy

This meta-analysis was conducted according to PRISMA guidelines. The English databases used for retrieval were PubMed, Cochrane, Web of Science, Embase, PsycINFO, PsycARTICLE, and the Chinese database was CNKI, CBM and WANFANG. The deadline is September 2020. In order to search OCD and BDNF rs6265 articles in the database more widely, no further restrictions were added. All published papers examined the association between OCD and the BDNF Val66Met polymorphism. The keywords “Brain-derived neurotrophic factor (BDNF),” “Brain Derived Neurotrophic Factor,” “Factor, Brain-Derived Neurotrophic,” “Neurotrophic Factor, Brain-Derived,” “BDNF,” “obsessive-compulsive disorder (OCD),” “Obsessive-Compulsive Neuroses,” “Val66Met,” “rs6265,” “G196A,” and “196G/A” were used to search the articles. We also examined all the references cited in the identified articles, as well as those cited in relevant review articles. The quality of the included studies was evaluated, and publication bias was evaluated.

### Data Extraction

From each enrolled article, data were extracted; including the name of the first author, year of publication, ethnicity of samples, diagnostic criteria of OCD, exclusion criteria, sample size (number of cases and controls), sample age, sex ratio, experimental design, severity of symptoms, and the available genotype information of the BDNF Val66Met polymorphism. To prevent potential errors, all data were extracted independently by the three authors, and a discussion within the research group resolved any disagreement.

### Data Analysis and Statistical Methods

The data analysis and statistical methods were based on previous research methods (9).

### Description of Studies Identified in Meta-Analysis

**Figure 1:**
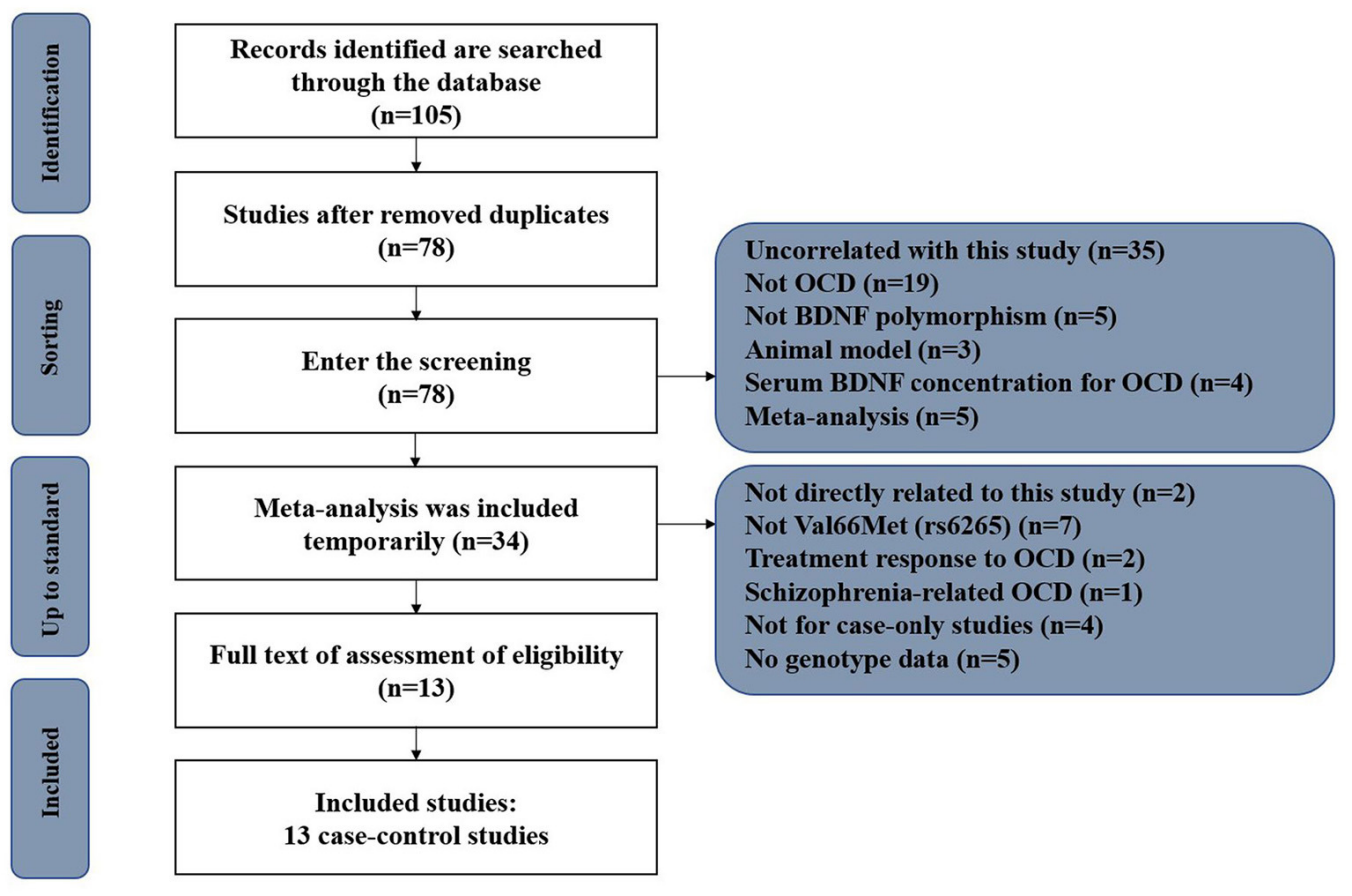
The process of independent studies for inclusion in this meta-analysis.

Through our search strategy, we identified 105 linked research papers with potential. After reading the abstracts and full compilations of the studies, 65 of them did not meet the inclusion criteria and were not able to be included (Figure 1). At the end, 13 available case-control studies were selected; including Wendland et al. (10), Hemmings et al. (11), Katerberg et al. (12), Wang et al. (13), Da Rocha et al. (14), Tükel et al. (15), Marquez et al. (16), Liu et al. (17), Hemmings et al. (18), Liu et al. (19), Wang et al. (20), Umehara et al. (21), and Taj et al. (22). One of the papers included separate data on two different groups of patients (12). After discussion within the research group, it was decided that two dependent sample data was still available, and therefore there were 13 articles in total included. Finally, all together there are 2765 OCD patients and 5558 psychiatrically healthy controls included. Table 1 lists the detailed characteristics of each study.

**Table 1:** Summary of studies examining the relationship between the BDNF val66met polymorphism and OCD.

**References**	**Race**	**Results**	**Genotype**						**Allele**			
			**Case (n)**			**Control (n)**			**Case (n)**		**Control (n)**	
			**Val/Val**	**Val/Met**	**Met/Met**	**Val/Val**	**Val/Met**	**Met/Met**	**Val**	**Met**	**Val**	**Met**
Wendland et al. (10)	Caucasian	No association	192	92	11	428	206	23	476	114	1,062	252
Hemmings et al. (11)	Caucasian	val66met variant associated with male EO OCD	73	33	6	95	43	2	179	45	233	47
Katerberg et al. I (12)	Caucasian	Val associated with sexual/religious obsessions	128	62	9	76	36	3	318	80	188	42
Katerberg et al. II (12)	Caucasian	Val associated with sexual/religious obsessions	132	75	13	352	166	17	339	101	870	200
Wang et al. (13)	Chinese Han	No association	31	76	41	24	51	19	138	158	99	89
Da Rocha et al. (14)	Caucasian	Met allele associated with OCD	83	33	11	98	21	8	199	55	217	37
Tükel et al. (15)	Caucasian	No association	23	54	23	29	51	30	100	100	109	111
Hemmings et al. (18)	Caucasian	No association	85	33	3	131	50	6	203	39	312	62
Liu et al. (17)	Chinese Han	No association	40	107	43	60	167	82	187	193	287	331
Márquez et al. (16)	NS	Val/Val associated with OCD	179	47	6	162	99	22	405	59	426	143
Liu et al. (19)	Chinese Han	val66met variant associated with OCD	101	166	54	110	221	105	368	274	431	421
Wang et al. (20)	Chinese Han	No association	31	76	41	24	54	21	138	158	102	96
Umehara et al. (21)	Japanese	No association	59	83	33	686	1,002	339	201	149	2,374	1,680
Taj et al. (22)	Indian	Met allele play a protective role in OCD	277	91	9	299	133	17	645	109	731	167

*BDNF, brain-derived neurotrophic factor; OCD, obsessive-compulsive disorder*.

## Results

Studies selected by the database searches examined the relationship between patients with OCD and Val66Met polymorphism distributions in the following populations: Chinese Han (*n* = 4), Caucasian (*n* = 7), Japanese (*n* = 1), Indian (*n* = 1) (Table 1). Polymerase chain reaction-based methods were used to identify the genotype and allele frequencies of both OCD patients and control subjects in all studies. The modified Newcastle-Ottawa scale (NOS) was used for quality assessment of the included studies, and the evaluation results are shown in Supplementary Table 1. The exclusion of articles with low scores (14) did not affect the results after statistical analysis, and detailed data can be seen in the Supplementary Figure 1 and Supplementary Table 2.

The studies included in Table 2 permitted our team to make planned comparisons of allele and genotype frequencies between OCD cases and controls. Prior studies have shown that the Val allele is linked with higher activity of the BDNF system when compared with the Met allele; therefore, we grouped the data for this meta-analysis according to the law of dominant or recessive modeling. We hypothesized that the Val genotype is required to confer susceptibility to OCD. The Stata software (Version15.0) was used to analyze the data.

**Table 2:** The clinical characteristics of the included studies.

**Characteristics**	**Sample size**	**Sex (male/female)**	**Age**	**Design**	**Exclusion criteria**	**Diagnosis**	**Measure to assess symptom severity**
Wendland et al. (10)	925	Not mentioned	≥18	Case-control study	Active schizophrenia or psychosis, severe mental retardation that does not permit an evaluation to characterize OCD, or OCD symptoms that occur exclusively in the context of depression were excluded	DSM-IV criteria	Y-BOCS
Hemmings et al. (11)	252	90/162	9–65	Case-control study	Not meet the DSM-IV criteria (APA 1994) for a primary diagnosis of OCD on the Structured Clinical Interview for Axis I disorders Patient Version were excluded.	DSM-IV criteria	Y-BOCS-SS
Katerberg et al. (12)	755	345/410	Not mentioned	Case-control study	Not meet the DSM-IV criteria were excluded.	DSM-IV criteria	Y-BOCS-SS
Katerberg et al. (12)	314	133/181	Not mentioned	Case-control study	Not meet the DSM-IV criteria were excluded.	DSM-IV criteria	Y-BOCS-SS
Wang et al. (13)	242	139/103	18–64	Case-control study	Severe physical illness, psychoactive substance abusers, serious suicide attempts, pregnant or lactating women were excluded.	DSM-IV criteria	Y-BOCS
Da Rocha et al. (14)	254	Not mentioned	Not mentioned	Case-control study	Not mentioned	DSM-IV criteria	Not mentioned
Tükel et al. (15)	210	80/130	17–50	Case-control study	Current psychiatric disorder other than OCD diagnosed with the SCID-I/CV, history of alcohol and/or drug abuse/dependence, neurological disease, pregnancy or lactation, medical disorders that may have a causal relationship with OCD	DSM-IV criteria	Y-BOCS
Márquez et al. (16)	515	260/255	Not mentioned	Case-control study	Not mentioned	DSM-IV-TR criteria	Y-BOCS
Liu et al. (17)	499	223/276	13–75	Case-control study	Severe physical disorder, abuse of psychoactive substances, pregnancy or lactation	DSM-IV criteria	Y-BOCS
Hemmings et al. (18)	322	134/188	12–72	Case-control study	History of neurological disease, schizophrenia, schizo-affective disorder, other psychotic conditions or a history of substance dependence, as determined from the interviews or previous medical records	DSM-IV criteria	Y-BOCS
Liu et al. (19)	747	414/333	≥18	Case-control study	Serious psychiatric diseases other than OCD or related family history, history of alcohol and/or drug abuse/dependence; any serious concomitant general medical condition or neurological disease except depression, phobia, anxiety or TS; history of medical disorders that may have a causal relationship with OCD; and pregnancy or lactation	DSM-IV criteria	Y-BOCS
Wang et al. (20)	247	143/104	16–64	Case-control study	Met any other DSM-IV axis I diagnosis; had any prior or current suicide attempts; were pregnant or lactating; or were in physical health such that they could not complete the study were excluded.	DSM-IV criteria	Y-BOCS
Umehara et al. (21)	2206	937/1265	Not mentioned	Case-control study	Patients comorbid with other axis I disorders were excluded.	DSM-IV criteria	Y-BOCS
Taj et al. (22)	826	Not mentioned	Not mentioned	Case-control study	Patients with co-morbid psychosis, bipolar disorder and mental retardation were excluded	DSM-IV criteria	Y-BOCS

*Axis I disorders (including dementia, substance abuse, dysthymia, panic disorder, obsessive-compulsive disorder, and generalized anxiety disorder); DSM-IV; the 4th edition of the diagnostic and statistical manual of mental disorder; DSM-IV-TR, DSM-IV-text version; Y-BOCS, Yale-Brown Obsessive Compulsive Scale; APA, American Psychological Association; Y-BOCS-SS, the Y-BOCS severity scale; SCID-I/CV, DSM-IV-TR Axis I disorders were examined using clinical protocols*.

**Figure 2:**
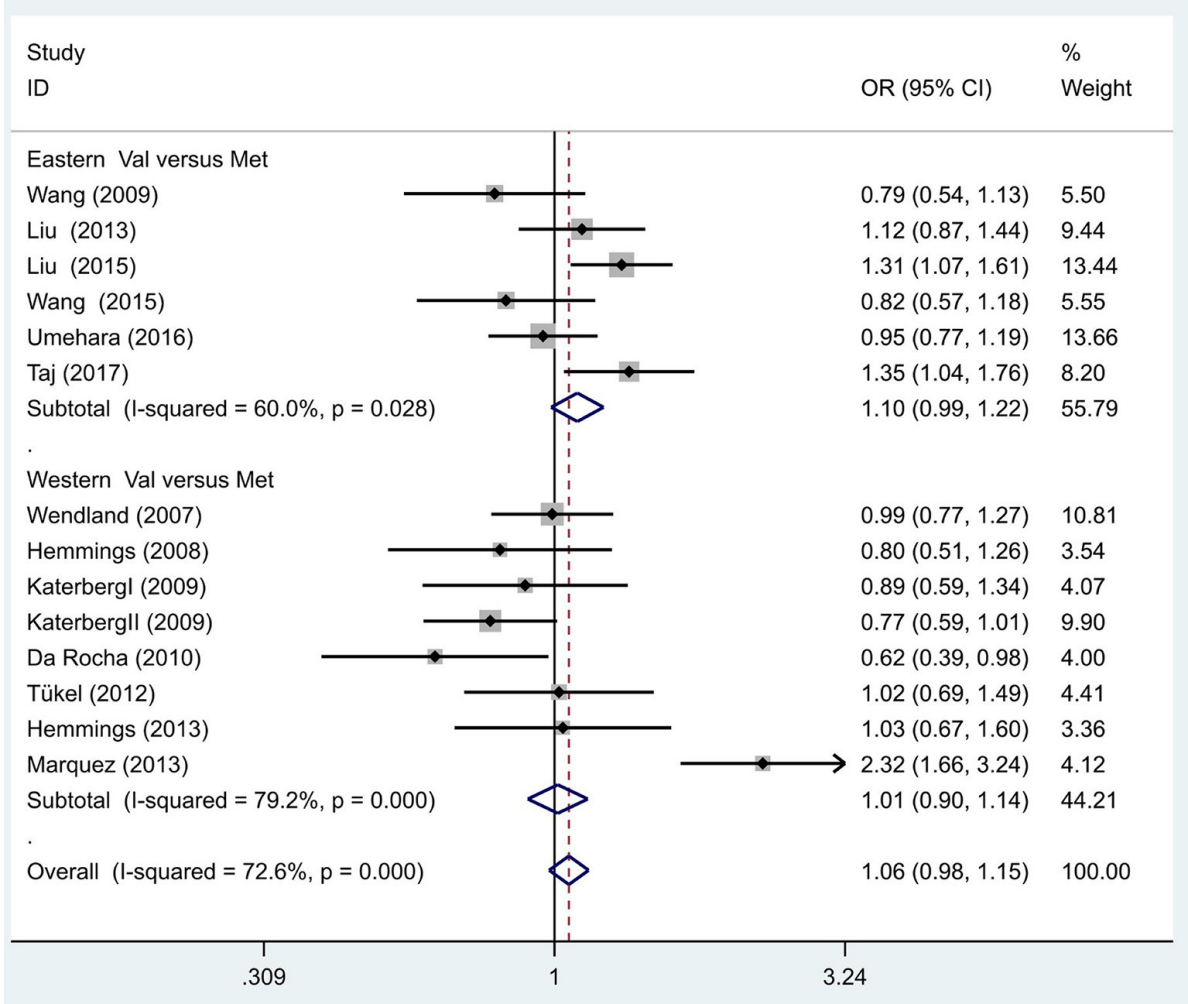
Results of the random-effects meta-analysis for the BDNF Val66Met allele (Val vs. Met) in OCD and control groups.

We also hypothesized that the Met allele is required to see a susceptibility to OCD. The between-study heterogeneity results hinted that the effect each individual study size had was statistically significant (*I*^2^ = 72.6%, *P* = 0.00; Figure 2). The pooled OR from these studies was 1.06 (95% CI = 0.98–1.15, z = 1.43, *P* = 0.15), denoting that there was no link between the Met allele and OCD.

Additionally, we hypothesized that in different populaces, the Met allele is linked with OCD, and proceeded to execute separate analyses in Eastern and Western populations. The between-study heterogeneity results hinted that in the Eastern population, the effect of study size was statistically significant (*I*^2^ = 60.0%, *P* = 0.03; Figure 2). The pooled OR from these studies was 1.10 (95% CI = 0.99–1.22, z =1.71, *P* = 0.09), hinting there was no link between the Met allele and OCD in the Eastern population. Similarly, no association was identified in the Western populations (OR = 1.01, 95% CI = 0.90–1.14, *z* = 0.22, *P* = 0.83; Figure 2).

**Figure 3:**
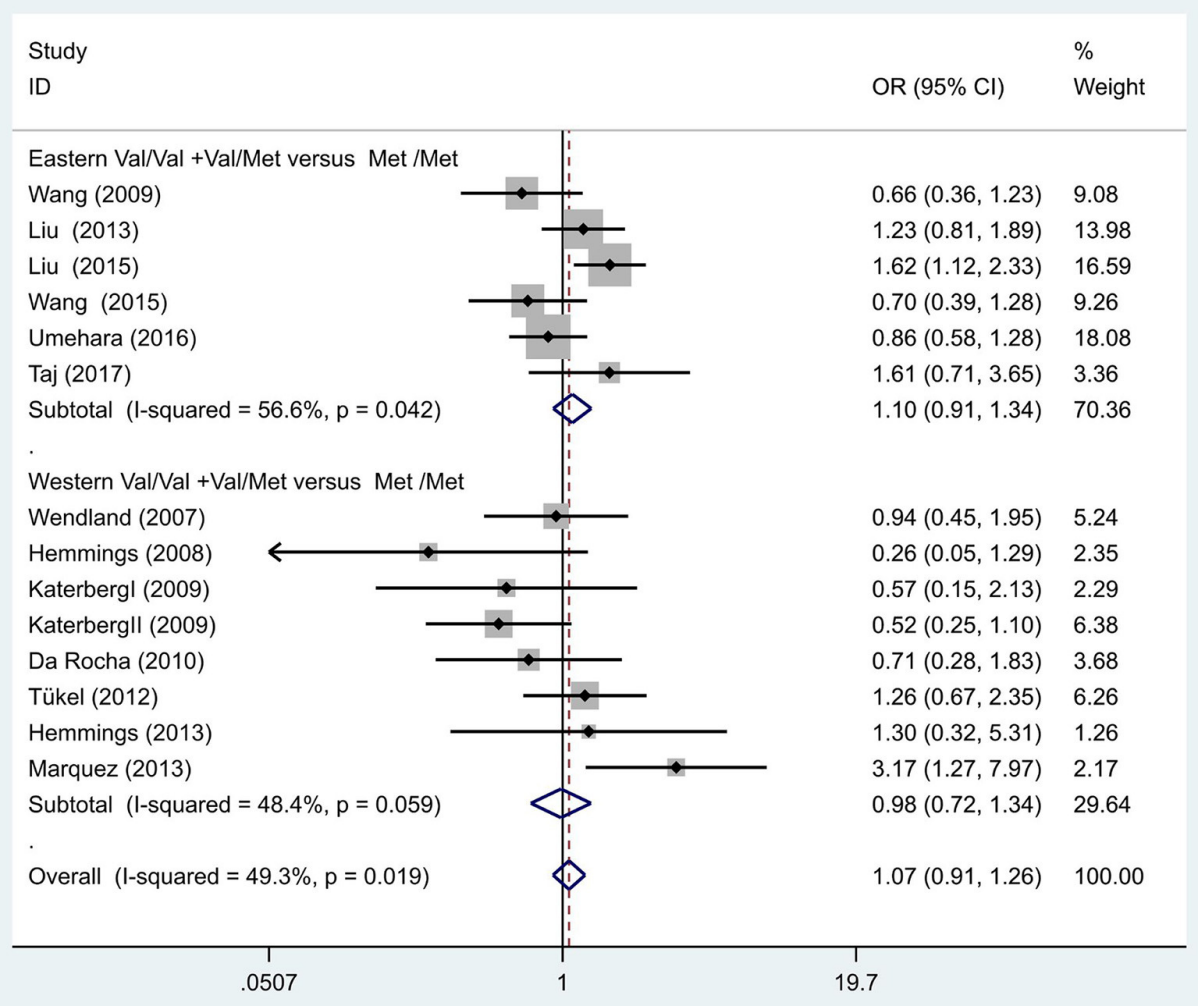
Results of the random-effects meta-analysis for the BDNF Val66Met genotype (Val/Val+Val/Met vs. Met/Met) in OCD and control groups.

**Figure 4:**
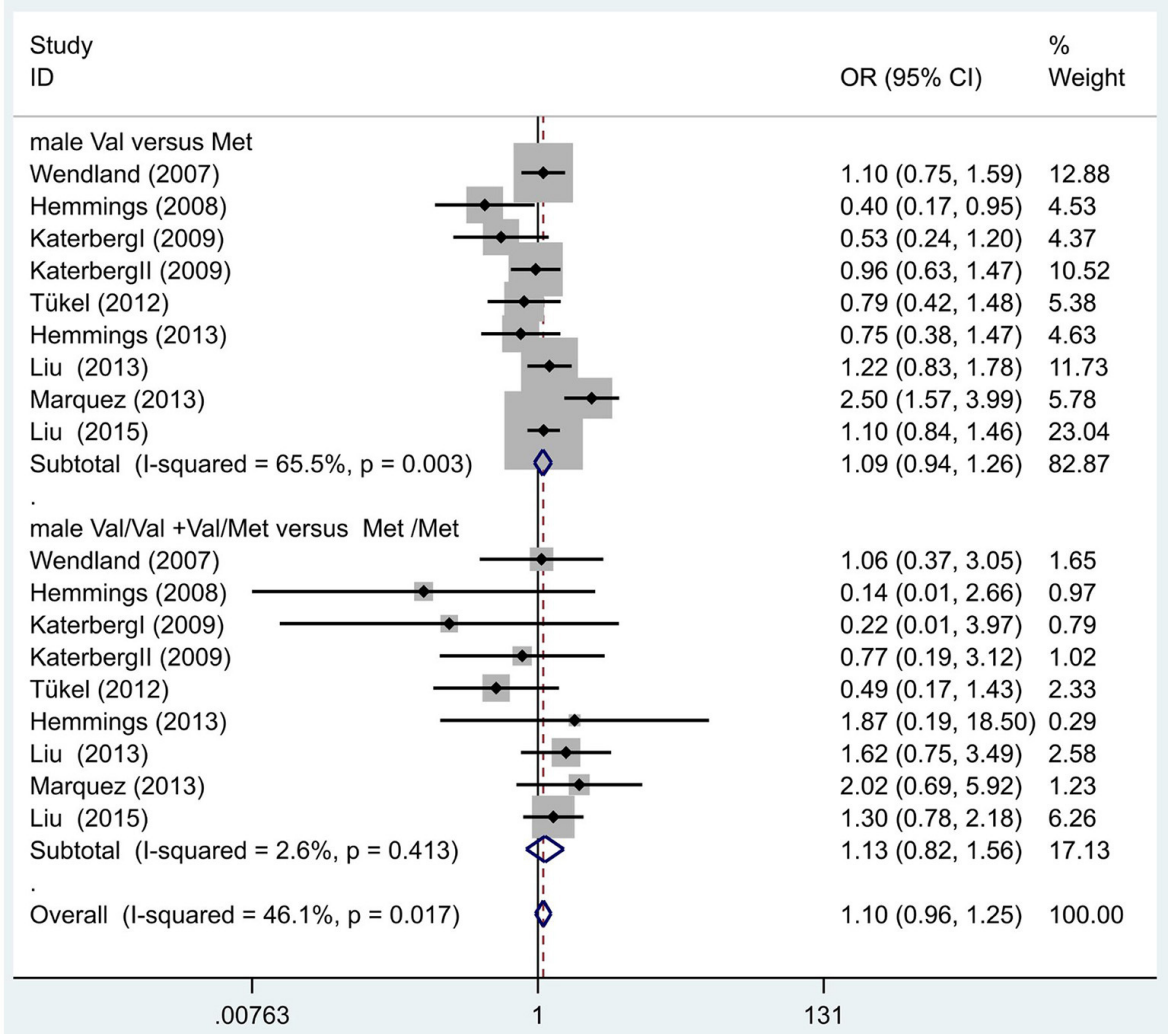
Results of the random-effects meta-analysis for the BDNF Val66Met Val carriers in male OCD and control groups, respectively.

**Figure 5:**
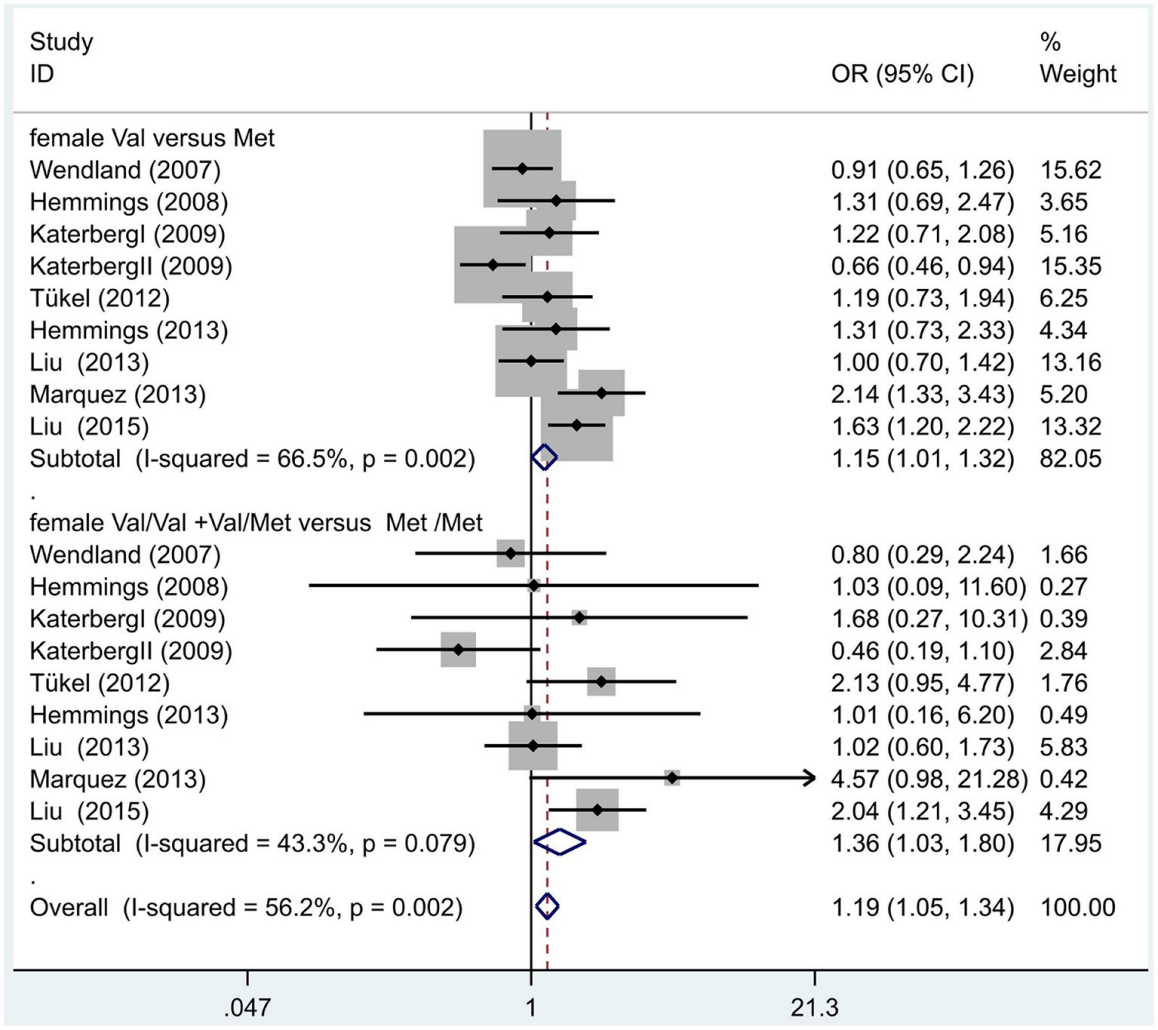
Results of the random-effects meta-analysis for the BDNF Val66Met Val carriers in female OCD and control groups.

Following our primary analysis, we hypothesized that the Met/Met genotype is a risk factor for OCD. It was suggested by the between-study heterogeneity results that the effect of sample size of each study was insignificant (*I*^2^ = 61.1%, *P* = 0.00). The pooled OR from these studies was 1.07 (95% CI = 1.00–1.14, *z* = 1.86, *P* = 0.06; Figure 3), indicating no association between the Met/Met genotype and OCD. Similarly, we also compared the Met/Met genotype vs. Val/Met +Val/Val alleles in the Eastern and Western populations, in order to explore their relationship with OCD further. We found that the results did not change in either the Eastern or Western population (OR = 1.10, 95% CI = 0.99–1.22, *z* = 1.71, *P* = 0.09; OR = 1.01, 95% CI = 0.90–1.14, *z* = 0.22, *P* = 0.83, respectively; Figure 3). Previous studies have demonstrated that sex-specific genetic architecture is a risk factor for OCD; thus, we compared Val *vs*. Met carriers in male and female groups. In gender-specific analysis, the female Val carriers pose as a risk factor for OCD (OR = 1.15, 95% CI = 1.01–1.32, *P* = 0.04 for allele; OR = 1.36, 95% CI = 1.03–1.80, *P* = 0.03 for genotype; Figure 4). No associations were found between male Val carriers and OCD (OR = 1.09, 95% CI = 0.94–1.26, *P* = 0.25 for allele; OR = 1.13, 95% CI = 0.84–1.56, *P* = 0.46 for genotype; Figure 5).

### Sensitivity Analyses

**Figure 6:**
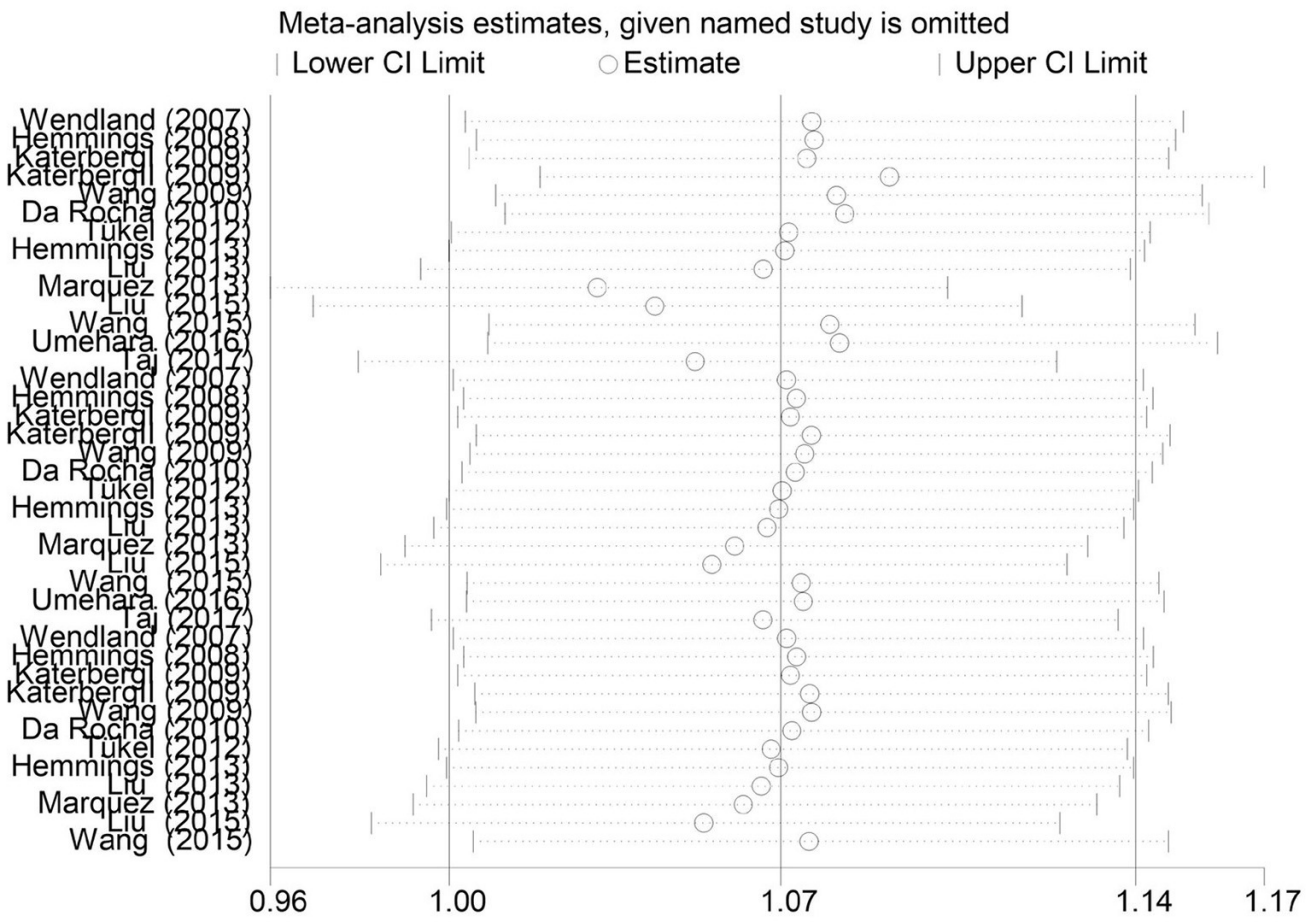
Results of sensitivity analysis.

The 13 studies we screened evaluated Val66Met polymorphisms using small sample sizes, indicating possible differences in genotype frequency and/or population stratification. We then performed a sensitivity analysis that excluded these studies one by one. However, the outcomes of the meta-analysis were not changed by sensitivity analysis (Figure 6).

### Publication Bias

The Begg-Mazumdar test for the BDNF Val66Met polymorphism demonstrated a low possibility of publication bias (*z* = −0.91, *P* = 0.363), and the Egger's test showed no significant results (t = −1.02, *P* = 0.86). Thus, we deduced there was no publication bias.

## Discussion

The currently updated meta-analysis is the most recent quantitative collection of available results from gene-psychiatric disorder association studies. The main findings of our studies indicate that the Val66Met polymorphism is not associated with OCD. Two prior meta-analyses have indicated that the Val66Met polymorphism is not related to OCD, which was in agreement with our findings (8, 20).

We first compared the gene gender-specific interaction effects in the etiology of OCD using a meta-analysis method. The main finding of this study is that in gender-specific analysis, the female Val carriers might be a risk factor for OCD (OR = 1.36, 95% CI = 1.03–1.80, *P* = 0.03 for genotype; OR = 1.21, 95% CI = 1.04–1.40, *P* = 0.01 for allele).

Sex differences in OCD etiology indicate a protective role of estrogens. It has been reported that male OCD patients display symptoms at a younger age and with more severe outcomes when compared to female patients (23), suggesting that sex differences may play a role in the progression of OCD. Furthermore, during the menstrual cycle, there are significant alternations in peripheral levels of BDNF, and BDNF levels have been strongly correlated with estradiol levels in females (24). In addition, Sohrabji et al. (25), demonstrated that through the estrogen response element's influence on the BDNF gene, the expression of BDNF could be regulated by estrogen. Even more, estrogen and some neurotrophins share certain signaling pathways (26), which explains how the BDNF gene is involved in the etiology of OCD. It also supports the notion that estrogens play a protective role in OCD development.

The results from Atmaca and Fouch (27, 28) demonstrated that subjects with OCD had smaller hippocampus volumes than controls. According to Egan et al. (29) and Hariri et al. (30), the Met allele is associated with abnormal hippocampal activation. Similarly, Egan has demonstrated that the BDNF Met allele significantly reduces the activity-dependent secretion of BDNF when compared with the Val allele (31). Therefore, the Met/Met genotype results in low BDNF protein activity and contributes to decreased hippocampus volumes, as seen in patients with OCD. Based on these findings and our results, we can speculate that the BDNF Val66Met polymorphism may confer susceptibility of hippocampus volumes in patients with OCD, and that is involved in the etiology of OCD.

Data that suggests there is no association of the Val66Met polymorphism with OCD is that the Met allele distributions range from 0 to 72% across different populations. This might explain the inconsistent results in different ethnic-backgrounds (32). The other possible explanation for the lack of association between the BDNF Val66Met polymorphism and OCD is the phenotypic heterogeneity of patients in the OCD sample. Selecting cases according to diagnostic criteria cannot assure an etiologically homogenous sample, because the diagnosis of OCD focuses on symptom-based criteria. Additionally, in the etiological mechanism behind OCD, the BDNF Val66Met polymorphism might not be directly involved, and may function instead to modulate disease development, such as symptom dimensions, age of onset, symptom severity, and family history of symptoms (10, 33–35). It is worth mentioning that the involvement of the BDNF gene may vary among distinct subtypes of OCD. Therefore, it is also possible that SNPs in the BDNF gene may only affect some of the symptoms of OCD (11, 12). Moreover, the Val66Met polymorphism may be in linkage disequilibrium with other functional polymorphisms of the BDNF gene, or in regulatory regions, such as BDNF C-270T, rs11030104, and rs10501087 (36–38). Combine these all together, it could be theorized that the rs6265 variant probably does not have a direct effect, but is in linkage disequilibrium with another causal variant. It can also be assumed that the linkage disequilibrium of rs6265, C-270T, rs11030104, and rs10501087 may vary among ethnic populations, thus contributing to the susceptibility to OCD. This may answer the inconsistency seen in the results from studies evaluating the association between the rs6265 polymorphism and OCD in different populaces.

A recent investigation highlighted that those individuals with obsessive–compulsive disorder (OCD) has comorbid lifetime diagnoses of major depressive disorder (MDD; rates <70%). Overlap exists in some common genetic variants (BDNF gene, for example) found across the affective/anxiety disorder spectrums (38). However, according to several meta-analysis of Val66Met polymorphism in the BDNF gene and MDD, the majority of them concluded that Val66Met polymorphism was not associated with MDD (39–41). Verhagen et al. suggested that the BDNF Val66Met polymorphism is of greater importance in the development of MDD in men than in women, while we reported that Val66Met polymorphism is of greater importance in the development of OCD in woman than in men. Future research into gender issues of these two diseases will be of interest (40).

Finally, in order to avoid the shortcomings of previous case-control and family-based studies in elucidating genes that contribute to psychiatric disorders, an endophenotype-based analysis has been recently adopted. This approach allows for a biological underpinning for diagnosis and classification, a net outcome to be established, and may help to identify the susceptibility of genes in the etiology of OCD. Future studies should adopt the endophenotype approach to improve ability to detect susceptibility loci in OCD in order to elucidate its etiology fully.

One major limitation for this study is that except for different genetic background, negative life events, personality, gender and environmental factors may also involve in the etiology of OCD. As it has been demonstrated that exposure to stressful life events before the onset of the illness seems to confer an increased risk for OCD. OCD probands have a significantly greater prevalence of ‘anxious' personality disorders in general and avoidant and obsessive-compulsive personality disorders as compared to controls (39–41), while environmental influences have shown a modest but significant impact on the OCD (42). Thus, in future study we combine BDNF val66met polymorphism, subclinical and environment factors interaction in the etiology of OCD. It has been demonstrated that gene–environment interactions are more likely to be involved in the etiology of OCD.

To conclude, our updated study suggests that the female BDNF Val66Met Val carriers may have an increased susceptibility to OCD.

## Data Availability Statement

The original contributions presented in the study are included in the article/Supplementary Material, further inquiries can be directed to the corresponding author/s.

## Author Contributions

XZ and HL conceived and designed the experiments. YS, NW, and EZ performed the experiments. QL and NW analyzed the data. YS wrote the article. All authors contributed to the article and approved the submitted version.

## Funding

A National Natural Science Foundation of China (No. 81371494) supported this study.

## Conflict of Interest

The authors declare that the research was conducted in the absence of any commercial or financial relationships that could be construed as a potential conflict of interest.

## Publisher's Note

All claims expressed in this article are solely those of the authors and do not necessarily represent those of their affiliated organizations, or those of the publisher, the editors and the reviewers. Any product that may be evaluated in this article, or claim that may be made by its manufacturer, is not guaranteed or endorsed by the publisher.
