# Supplementary material for: The Brain-Derived Neurotrophic Factor Val66Met Polymorphism Is Associated With Female Obsessive-Compulsive Disorder: An Updated Meta-Analysis of 2765 Obsessive-Compulsive Disorder Cases and 5558 Controls
Source: Front Psychiatry. 2022 Jan 12;12:685041. doi: 10.3389/fpsyt.2021.685041 (PMC8791441; doi:10.3389/fpsyt.2021.685041)

Supplementary Table 1. The Newcastle-Ottawa Scale (NOS) for assessing the quality of studies

| Study | Definition of cases | Representative  Of cases | Selection of controls | Definition of controls | Comparability of cases and controls on the basis of the design or analysis | Ascertainment of exposure | Same method of ascertainment for cases and controls | Non-response  rate | Total scores |
| --- | --- | --- | --- | --- | --- | --- | --- | --- | --- |
| Wendland et al.(2007) | * | — | * | — | * | * | * | * | 6 |
| Hemmings et al.(2008) | * | * | * | _ | * | * | * | * | 7 |
| katerberg et al.Ⅰ(2009) | * | — | * | — | * | * | * | * | 6 |
| katerberg et al.Ⅱ(2009) | * | — | * | — | ** | * | * | * | 7 |
| Wang et al.(2009) | * | * | — | * | * | * | * | * | 7 |
| Da Rocha et al.(2010) | — | — | — | * | * | * | * | — | 4 |
| Tükel et al.(2012) | * | * | * | * | ** | * | * | * | 9 |
| Hemmings et al.(2013) | * | * | — | * | ** | * | * | * | 8 |
| liu et al.(2013) | * | * | * | — | ** | * | * | * | 8 |
| Marquez et al.(2013) | * | * | * | * | * | * | * | * | 8 |
| Liu et al.(2015) | * | * | * | * | * | * | * | * | 8 |
| Wang et al.(2015) | * | * | * | * | * | * | * | * | 8 |
| Umehara et al.(2016) | * | * | — | * | * | * | * | * | 7 |
| Taj et al.(2017) | * | * | — | * | * | * | * | * | 7 |
| Average star (means± SD) |  |  |  |  |  |  |  |  | 7.14±1.23 |

Supplementary Figure1. Forest plot for Val versus Met (allelic model) in total and Western population

Supplementary Table 2. Overall and stratified analyses of the BDNF Val66Met polymorphism on OCD risk.


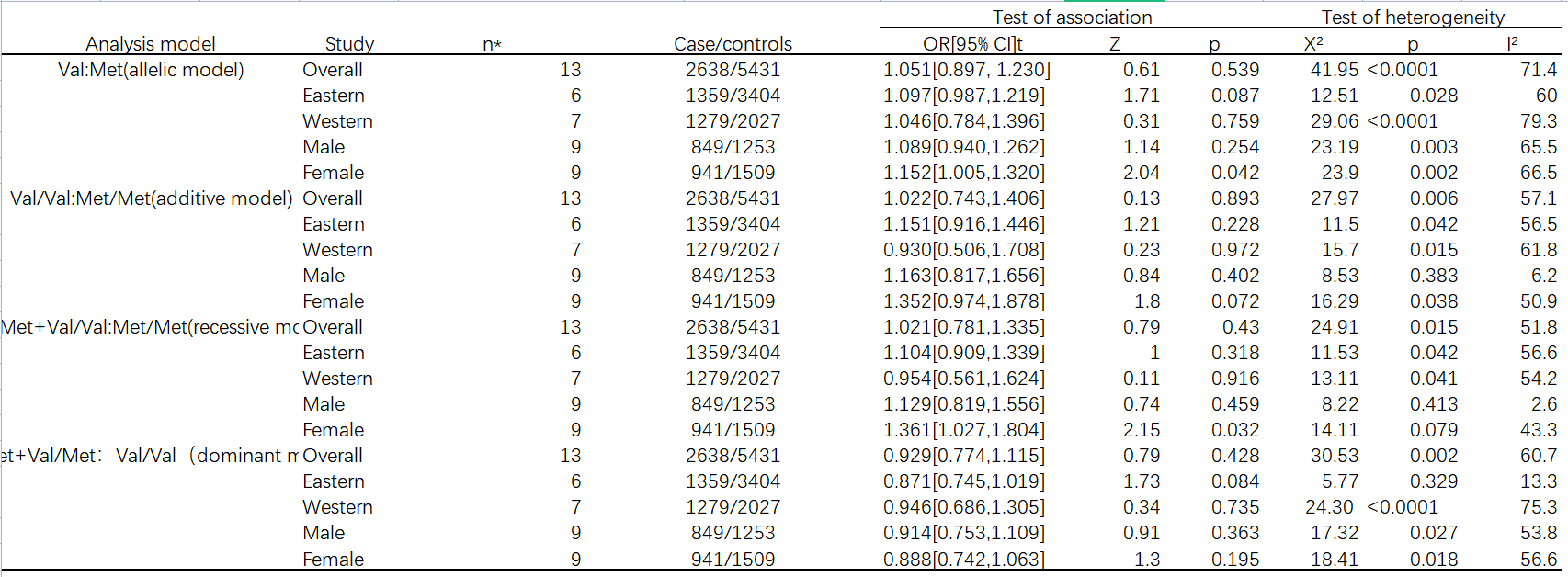

Supplement: Supplementary file 1 [file Table_1.DOCX]
